# Chronic Exposure of Human Endothelial Progenitor Cells to Diabetic Condition Abolished the Regulated Kinetics Activity of Exosomes

**Published:** 2018

**Authors:** Mehdi Hassanpour, Omid Cheraghi, Belal Brazvan, Amirataollah Hiradfar, Nasser Aghamohammadzadeh, Reza Rahbarghazi, Mohammad Nouri

**Affiliations:** a *Department of Clinical Biochemistry and Laboratory Medicine, Tabriz University of Medical Sciences, Tabriz, Iran. *; b *Stem Cell Research Center, Tabriz University of Medical Science, Tabriz, Iran. *; c *Department of Biochemistry, Faculty of Biological Sciences, Tarbiat Modares University, Tehran, Iran.*; d *Pediatric Health Research Center, Tabriz University of Medical Sciences, Tabriz, Iran. *; e *Endocrine and Metabolism Section, Department of Internal Medicine, Tabriz University of Medical Sciences, Tabriz, Iran.*; 1 ^1^ R.R and M.M. contributed equally to this work

**Keywords:** Endothelial progenitor cells, Diabetes, Exosome, Kinetics

## Abstract

By virtue of lifestyle change, incidence of diabetes mellitus type 2 is increasingly being raised with different up-surging pathologies. It was showed that endothelial progenitor cells (EPCs) were disqualified in neo-angiogenesis induction. Besides, to an aborted differentiation property, malfunctioned paracrine activities worsen off vascular abnormality. Nano-scaled exosomes play essential roles in reciprocal cell-cell crosstalk via bioactive molecules. To address the effect of diabetic serum on exosome secretion capacity, EPCs were exposed to diabetic condition for seven days. In addition to *in-vitro* tubulogenesis, migration and LDL uptake assessment, exosome release capacity, and expression profiles of three genes participating in exosome kinetics, including CD63, Alix and Rab27a, revealed by Real-time PCR method. Data showed diabetic sera not only abolished the *in-vitro* tubulogenesis, migration and LDL uptake properties but also decreased exosome release and expression of related genes. This study sheds lights on the adverse effect of diabetic condition on exosome kinetics in EPCs.

## Introduction

Because of changes in modern life style patterns attributing to lack of sufficient mobility, exercise and unfit obesity, the incidence of diabetes mellitus type 2 (T2DM) is increasingly being raised worldwide (1). Uncontrolled alterations in endothelial cells (ECs) function, a major cell component of furnishing luminal surface, emerge soon due to dynamic complexity of diabetes causing the occurrence of clinical signs associated with vascular insufficiency in various tissues (2). For example, lack of an appropriate wound healing and imbalanced angiogenesis process influenced by delayed or impaired vessels growth provokes *per se* the continuous vicious cycle of abnormality (3).

It was previously found that EC lineage and peculiarly Endothelial Progenitor Cells (EPCs), first identified and described by Asahara and colleagues, actively recruited and incorporated into the dynamic angiogenic site by a sophisticated sequence of multiple signaling occurrences (4,5). Although, no exclusive meaningful markers of EPCs had not yet been determined but a great body of experiments confirmed the expression form of cell surface markers such as CD133, CD34, Vascular Endothelial Growth Factor Receptor-2 (VEGFR-2; also termed Flk-1), Tie-2, c-Kit and CXCR_4_ (6). *In-vitro* experiments showed two distinct chronological order of EPC population typified by formed colonies, namely early, and late outgrowth colonies with different cell morphology of spindle-shaped and cobblestone like appearance, respectively (7). Of note, extensive capacity for giving rise to various lineages of mature cells peculiarly ECs, accompanied with paracrine activity and the maintenance of endothelium hemostasis, play an essential role in therapeutic effect exerted by EPCs (7). Additionally, in response to different stimuli such as ischemia, cytokines, growth factors, and physical exercise, EPCs easily transmigrate from bone marrow niche into systemic circulation, by then nominated as circulating endothelial progenitor cells, which further polarized to the damaged endothelium (8). An exciting result of pre-diabetic and diabetic conditions reveled lower levels of circulating CD45^dim^CD34^+^KDR^+^ and CD45^dim^CD133^+^KDR^+ ^EPCs inversely correlated with HbA1c content (9).

Similar to different cells (10), a part of paracrine secretion activity in EPCs is routinely managed by exosomes biogenesis (11). These membrane-restricted nano-scale extracellular vesicles ranging from 30 to 100 nm emanate from the inner budding of the cell membranes followed by making of multi vesicular bodies (MVBs) (10). Smart package of exosomes encompassing a vast array of specific content, such as lipids, proteins, nucleic acids including mRNAs and microRNAs and their thereof, are demonstrative to their cellular origin and thereby interplay as a biological bridge between giver and receiver cells through body fluids (12). 

By virtue of endosomal origin, exosomes have been specific unified proteins in their disposal to control dynamic kinetics of machinery transport of intracellular trafficking, fusion and abscission (13). Based on different works, an extensive panel of critical factors, separately or in association with together, such as CD63, TSG101, Alix, HSP70, CD9, Mfge8, Rab27a, b family, *etc*. was found to actively participate in exosome biogenesis (14). Effective marker termed as PDCD6IP, known also as Alix, participates in the budding, sorting and abscission of formed exosomes (14, 15). Another relevant protein from tetraspanin family, such as CD63, has been evident in budding of the limiting endosomal membrane and fusion to plasma membrane (16). In line with above-mentioned information, Ostrowski and colleagues previously acclaimed that GTPases of the Rab family especially Rab27a or Rab27b initiated MVBs docking to the cell plasma membrane (17).

In addition to impaired function of EPC in terms of aberrant proliferation, adhesion and incorporation into vascular beds, and concurrent diminished circulating levels of EPCs occurred under diabetic status (9), it seems that the secretory capacity of exposed EPCs to diabetic condition was also decreased (18). However, there is a little information regarding to the potent side effects of abnormal diabetic condition on the exosome secretion capacity of EPCs. In this study, we further evaluated the hypothesis, whether a diabetic state could reduce the dynamics biogenesis, trafficking, and fusion of exosome in human EPCs (hEPCs) via CD63-Alix-Rab27a axis. To attain this aim, we exposed healthy isolated hEPCs to sera from newly-diagnosed diabetic subjects over a period of seven days and results compared with parallel control. 

## Experimental

*Provision of diabetic and non-diabetic sera*

In current experiment, 10 diabetic sera were supplied from newly-diagnosed diabetic males aged more than 40 years old without any drug consumption history. Non-diabetic sera were also provided from age-matched individuals. All of the participants in both groups were enrolled into current experiment subjected to a convenient metabolic screening (Table 1). Informed consent was endorsed by all of volunteers enrolled to this study. All procedures performed through the studies, involving human participants, were in accordance with the local research ethic committee of Tabriz University of Medical Sciences and ethical principles of the declaration of Helsinki.

*hEPCs isolation and expansion protocol*

Bone marrow aspirates of healthy volunteers, ranging from 3 to 30 years old, presented to the clinical laboratory of Shahid Ghazi hospital and Children hospital, an affiliated hospitals to Tabriz University of Medical Sciences, were exploited in the current study. Informed consent was obtained prior subjecting each patient to current experiment. In short, a sample volume of 2 ml blood remained after diagnostic tests was used for the isolation of mononuclear cells (MNCs) in Ficoll density gradient according to the manufacturer’s protocols and heparin (1,000 IU/mL) used as an anticoagulant. Blood was further diluted 1:3 (v/v) with phosphate buffered saline solution (PBS) and equal sample of diluted blood overlaid to Ficoll-hypaque solution (Sigma), centrifuged 20 min at 400* g* at 5 °C. Thereafter, the collected bone marrow-derived MNCs at the interface between the plasma and the underlain Ficoll solution was harvested, washed twice with PBS and resuspended in the complete EGM-2 medium (Promocell, Cat No: C-39211) and layered plates coated by fibronectin (1 µg/mL; Promocell). After 4-day incubation, the exhausted supernatant containing non-adherent cells and any undesirable cell phenotypes were replenished by fresh media.

*The confirmation of EPC phenotype by clonogenicity assay*

To determine whether the EPCs pre-expanded on a fibronectin-coated surface maintained clonogenicity property during 7 days prior to an experimental procedure, a methylcellulose semi-solid colony formation assays was performed in according to our previous work with some modification (7). An initial density of 5 × 10^4 ^of hEPCs pre-cultured on the fibronectin substrate were detached and maintained in methylcellulose medium containing EGM™-2 BulletKit™ and maintained for one weeks.

*Flow cytometric immunophenotypic evaluation of isolated hEPCs*

 On day 7, surface-markers of fibronectin-adherent cells were evaluated via flow cytometric analysis by using a panel of antibodies directed against cell surface markers including FITC-conjugated anti-human CD133 (Miltenyi Biotech), Tie2 (Abcam), CD117 (ebioscience), CD45 (ebioscience), as well as PE-conjugated anti-human CD33 (ebioscience), VEGFR2 (Abcam), CD14 and CD34 (ebioscience). Single cell suspension was harvested after treatment by 0.025% Trypsin–EDTA solution (Gibco), blocked with 1% BSA for 20 min, and incubated with antibodies according to manufacturer′s instruction. To exclude background staining, appropriate isotype control antibodies ware used. After twice washing by PBS, the cell suspension mixed with equal volume of 4% paraformaldehyde and analyzed by BD FACSCalibur flow cytometer. Finally, the raw data was processed using FlowJo software ver. 7.6.1. 

*In-vitro cytotoxicity assay*

Cytotoxicity activity of hEPCs induced by sera of diabetes mellitus type 2 patients or controls were evaluated by MTT [3-(4, 5-dimethyl thiazol-2-yl) 2, 5-diphenyl-tetrazolium bromide] assay. In short, a 200 µL of EGM-2 medium supplemented with 2% FBS containing a 5 × 10^5^ hEPCs were plated in each well of 96-well plates and kept for 24 to 48 h. Thereafter, the supernatant was discarded and replaced by a 200 µL EGM-2 medium containing 10% diabetic or non-diabetic serum and incubated for next 7 days. Afterward, the media were discarded and 50 µL of MTT solution (5mg/mL; Sigma) added to each well. After 4 h incubation time, 200 µL (Dimethyl Sulfoxide) DMSO solution was layered and the absorbance determined at 490 nm with a microplate reader (Bio-Tek). Taken data were originated from six pooled diabetic and non-diabetic sera performed in three independent experiment sets of octuplicate. The possible cytotoxic/cytostatic effect of diabetic sera was expressed as the relative viability (% control). 

**Table 1:** Metabolic screening of diabetic and non-diabetic individuals. The values were expressed in mean ± SD from 10 healthy and diabetic individuals

	**Fasting blood sugar (mg/dL)**	**Blood sugar (mg/dL)**	**Cholesterol (mg/dL)**	**Triglyceride** **(mg/dL)**	**LDL (mg/dL)**	**HDL** **(mg/dL)**	**Creatinine** **(mg/dL)**	**HbA**_1_ **c (%)**
Healthy serum	88.2±4.5	122.2±6.1	181.3±5.4	85.3±7.6	98±5.2	49.8±5.2	1±0.1	5.1±0.2
Diabetic serum	153±9.5	211.5±10.6	246±7.6	200±9.8	154.8± 11.1	39.2±5.2	1.1±0.1	7.9±1.4

**Table 2:** The list of primers used in real-time PCR

**Candidate genes**	**Forward**	**Reverse**
CD63	CCCAGCTGTCTGCACAGTCGG	CAGAGAAGCGGACGAGGTGGG
Alix (PDCD6IP)	CTGGAAGGATGCTTTCGATAAAGG	AGGCTGCACAATTGAACAACAC
Rab27a	GAAAGAGGAGGAAGCCATAGCAC	CATGACCATTTGATCGCACCAC
β-actin	TCCCTGGAGAAGAGCTACG	GTAGTTTCGTGGATGCCACA

**Figure 1:**
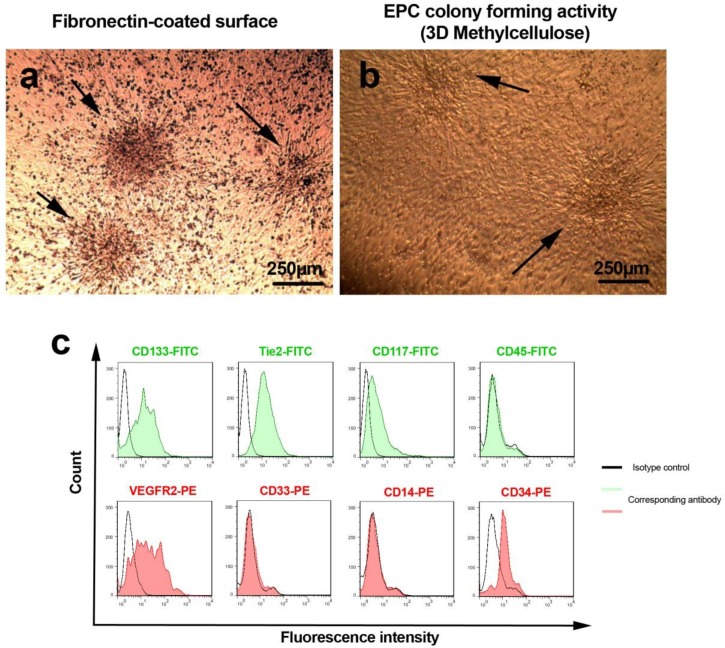
Morphological characteristics of 7-day pre-cultured hEPCs on fibronectin substrate (**A**). As shown here, three colony-forming units of human endothelial progenitor cells were revealed during the first 7 day of plating (panel **A**; black arrows). In addition two hEPC colonies were shown in 3D methylcellulose semi-solid medium to confirm the stability of stemness (panel B; black arrow). Both negative and positive human endothelial progenitor cells markers were analyzed by using flow cytometric assay and presented in histogram (C).

**Figure 2:**
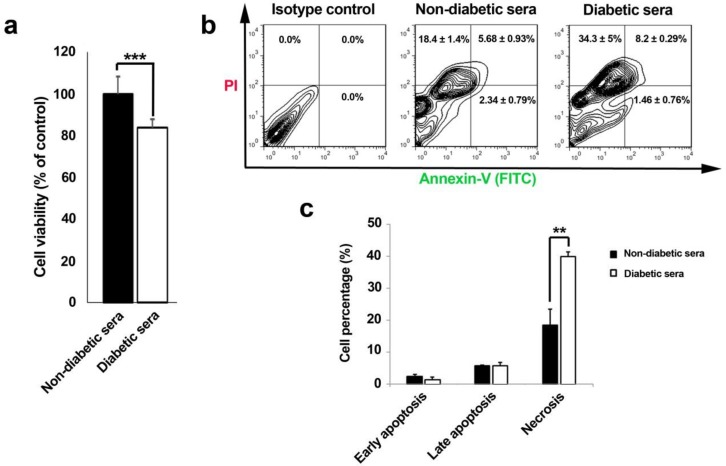
Effect of diabetic and non-diabetic sera on cell viability rate (% control) of human endothelial progenitor cells after 7-day incubation (**A-C**). Cell viability rate was significantly decreased in diabetic sera exposed cells (**A**). Annexin-V/PI double staining assay showed an increased percentage of necrotic cell rather than apoptotic changes (**B** and **C**). Data were presented as means ± SD. Statistical analysis was performed using student's *t*-test. *p < 0.05, **p < 0.01, ***p < 0.001.

**Figure 3:**
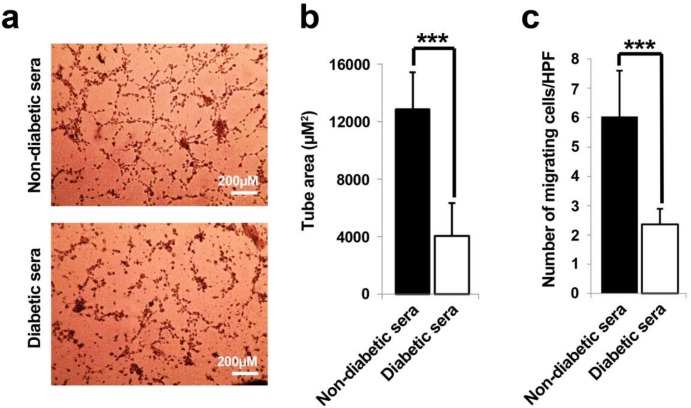
Representative illustration of *in-vitro* tubulogenesis and migration assays (**A**-**C**). It was notified that the diabetic sera exerted drastic detrimental effect on endothelial progenitor cells tube formation capacity as compared to control subjects (**A** and **B**). A low number of migrated cells were determined in diabetic sera-exposed cell in trans-well migration assay (**C**). Data were presented as means ± SD. Statistical analysis was performed using student's t-test. *p < 0.05, **p < 0.01, ***p < 0.001.

**Figure 4:**
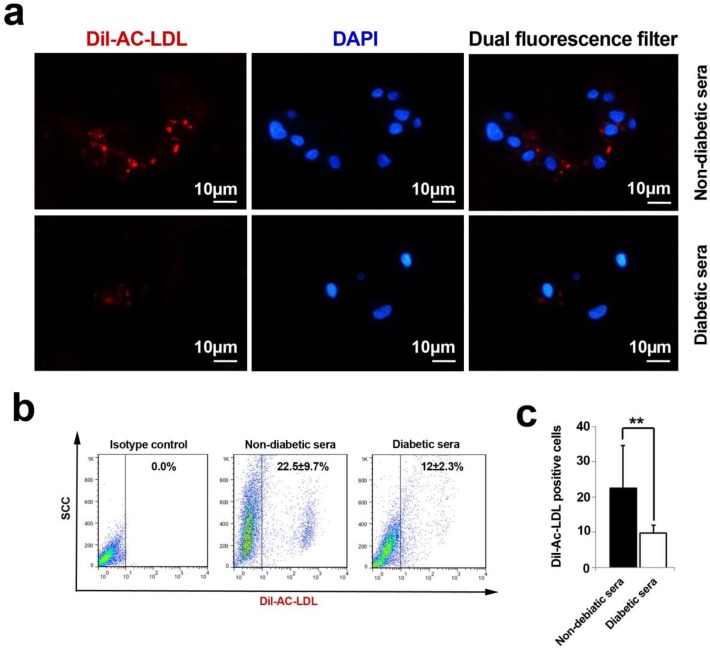
An analysis of Ac-LDL uptake capacity by immunofluorescence and flow cytometry techniques (**A**-**C**). The cells lost their ability to uptake Ac-LDL under diabetic condition. Both immunofluorescence imaging (**A**) and flow cytometric analysis (**B**) confirmed a vivid decline in the percent of fluorescent tag cells (**C**). Data were presented as means ± SD. Statistical analysis was performed using student's t-test. * *p *< 0.05, **p < 0.01, ***p < 0.001.

**Figure 5:**
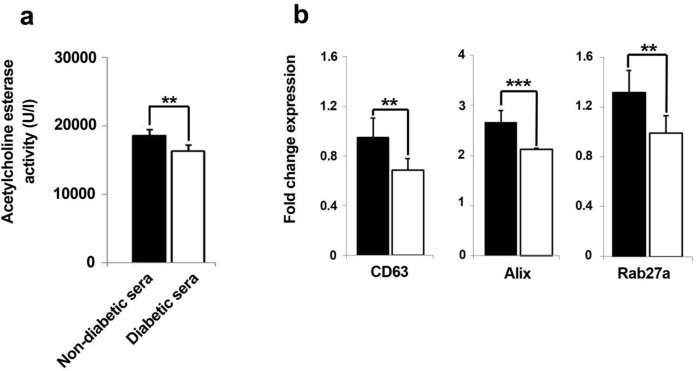
The levels of released exosomes were quantified by measuring of exosome-associated acetylcholine esterase activity in the conditioned media from two set of groups (**A**). Data represent that amount of exosome profoundly decreased in diabetic sera-treated hEPCs (**A**). Real-time PCR analysis showed significant down-regulation of exosome biomarker genes after hEPCs being-treated with diabetic sera (**B**). Data were presented as means ± SD. Statistical analysis was performed using student's t-test. *p < 0.05, **p < 0.01, ***p < 0.001.

*Apoptosis and necrosis detection by Annexin-V/Propidium iodide assay*

To pinpoint the possible underlying cell cytotoxicity mechanism in 7-day cultured hEPCs under normal and diabetic conditions, a convenient double Annexin-V/Propidium iodide assay was performed. Briefly, cells were trypsinized, washed twice with PBS, collected, resuspended in the 100 μL binding buffer and kept for 30 min at RT. Afterwards, 5 μL FITC-conjugated Annexin-V was added and incubated for 15 min at RT. Next, they were incubated with 5 μL of propidium iodide (PI) solution for 15 min. ultimately, cells were analyzed by FACSCalibur (BD Bioscience) system and data were analyzed with FlowJo software ver. 7.6.1. 

*In-vitro tubulogenesis assay*

*In-vitro* tube formation assay was performed by using growth factor-depleted Matrigel (Corning) in the 3D culture model. Briefly, an equal volume of pre-chilled Matrigel was diluted with M199 medium. Then, 48 well-plates were coated with 100 μL Matrigel per well kept at 37 °C for 30 min to be solidified. hEPCs being-exposed to diabetic and control sera were plated at an initial density of 2 × 10^4 ^cells on Matrigel substrate. After 24 h of incubation, the formed tube-like structures were imaged at using an inverted microscope. To further quantitate the *in-vitro* angiogenesis, enclosed area of 5 random serial microscopic fields per each well was analyzed in µm^2^ using an image-analyzing software package (Image J software, NIH). The mean number of tubular area ± SD was indicated for each condition. 

*Transwell migration assay*

To well-compare the migratory ability of hEPC under both conditions, an *in-vitro* Transwell migration assay was performed by using Transwell inserts of 24 well plates with 8 μm pore size. A cell density of 5 × 10^4^ in 200 μL M199 medium supplemented with %2 FBS was added to apical insert while 750 µL M199 medium devoid of FBS with 20 ng/mL basic fibroblast growth factor (bFGF; Sigma) was located in basolateral space. 24 h after incubation, the migrated cells at bottom surfaces were counted six random fields per well. All experimentations were performed in triplicate.

*Function Analysis of EPCs via Low-density lipoprotein uptake*

The uptake of acetylated low-density lipoprotein (Ac-LDL) was assessed on 7-day cultured EPCs under diabetic condition as previously described (7). In brief, an initial number of 10^5 ^EPCs was plated on each well of 8-well chamber slide (Cat No: 30108; SPL) and exposed to diabetic sera. Thereafter, the cells were incubated with 100 µL EGM-2 containing 10 µg/mL of 1, 1′-dioctadecyl-3, 3, 3′, 3′-tetramethyl-indocarbocyanine perchlorate (Dil)-labeled Ac-LDL (Cat No: J65597; Alfa Aesar) at 37 °C for 4 h. Afterwards, they were washed twice with PBS and fixed with 4% paraformaldehyde for 10 min at RT. To nuclear counterstaining, 1 µg/mL of 4′, 6-diamidino-2-phenylindole (DAPI) solution was used. Finally, the Dil-positive cells were imaged by using an inverted fluorescence microscopy (Olympus). In addition, a flow cytometric analysis was also assessed to precise determination of Dil-Ac-LDL^+^ cells. 

*Determination of supernatant exosome content based on acetylcholine esterase activity*

We also analyzed the presence of cells being-exposed by normal and diabetic conditions via acetylcholine esterase activity, as an exosome marker protein, by spectrophotometric assay. First, EPCs of 7-day exposed to diabetic or non-diabetic conditions were washed three times with PBS and incubated with M-199 devoid of growth factor and FBS for 48 h. Thereafter, a volume of 20 µL of each groups was mixed with 500 µL buffer solution containing 75mM pyrophosphate and 2 mM potassium hexacyanoferrate for 5 min at RT (Cat No: BXC0801; biorexfars). Then, 100 µL of s-butyrylthiocholine iodide was added and final absorbance read at 405 nm during three different intervals. Finally, the choline esterase activity was calculated by following formula;

*Activity (U/l) = ∆Abs/min × 65800*

*Quantitative real-time PCR (qRT-PCR)*

We also determined the stimulatory/inhibitory effect of diabetic sera on exosome biogenesis through 7 days. Seven days after exposure to diabetic or non-diabetic conditions, the total content RNA of hEPCs was isolated. Briefly, the cell pellet was washed with PBS, and then 1 mL of RNX-plus buffer (Cat No: MR7713C; CinnaGen) added and further vortexed to homogenize the clumps. Thereafter, 200 μL of chloroform was added and centrifuged at 12000 rpm for 15 min at 5 °C. Next, the upper phase containing RNA and DNA was mixed with an equal volume of isopropanol (Sigma) and incubated at 4 °C for 15 min. After centrifugation, the resultant supernatant was discarded and pellet was dissolved in 1 mL of 75% ethanol solution. Finally, the total content of harvested RNA, in 50 µL DEPC treated water, determined by a NanoDrop (Thermo Scientific). To exclude a possible genomic DNA contamination, RNA solutions were treated with DNase1 kit (Cat No: en0521; Fermentaz) according to manufacturer′s instruction. 

Prior to real-time PCR assay, purified total RNA was reversely transcribed. Further, the qRT-PCR reaction was performed with the synthesized cDNA, SYBR premix Ex Taq kit (Cat No: RR820L; TaKaRa) and candidate gene primers according to the manufacturer’s protocol. The qRT-PCR was launched by a Rotor Gene Corbett System (Model: R080873). The raw data was analyzed by convenient Pfaffl method with normalization to housekeeping gene β-actin. The experiment was conducted in triplicate. The forward and reverse primer sequences for human CD63, Alix and Rab27a were outlined in Table 2.

*The morphological and immunophenotypic features of isolated hEPC*

Three days after MNCs plating, the adherent EPCs initiated to generate primary clusters that reached to their maximum size at day 7 and thereby cells became more spindle-shaped and elongated as described by A sahara *et al* (19) (Figure 1A). We also defined that EPCs pre-cultured on fibronectin maintained their clonogenicity even at day 14 in methylcellulose semisolid media (Figure 1B). The flow cytometric analysis confirmed the existence of ECP-related antigens such as CD133, Tie-2, CD117, VEGFR-2, and CD34 while nonspecific markers such as myeloid and monocytic surface antigens including CD14, CD45, and CD33 were not evident in current experiment (Figure 1C).

*The priming of EPCs with diabetic sera induced cell mortality mainly by necrotic changes*

Corroborating to our MTT results, cell viability rate drastically diminished through 7-day incubation of EPCs with diabetic serum (Figure 2A). Interestingly, it was also notified that outstanding shifts in cell viability happened by means of necrotic changes during diabetic serum incubation as compared to cells under normal conditions (Figure 2B and C). Therefore, regarding to time and concentration of diabetic sera applied here, necrosis was illuminated as the most prominent cell cytotoxic effect.

*EPCs in-vitro tubulogenesis was inhibited after being in diabetic condition*

Matrigel tube formation assay was exploited to evaluate the effects of diabetic and non-diabetic sera on EPCs angiogenic properties. Twenty four hours after seeding of 7-day treated cells on Matrigel substrate; the development of tubular-like structures with formed lumens was assessed (Figure 3A and B). EPCs under healthy non-diabetic sera, immediately migrated toward together, elongated with reciprocal polarization to form tube area (Figure 3A). In contrary, the area of vascular-like network area was remarkably diminished in diabetic-treated cells as compared to control (*p* = 0.0004).

*The chemotactic response of EPCs was obviously decreased toward bFGF stimulation under diabetic condition*

Next, the effect of both diabetic and non-diabetic healthy sera was examined on EPCs migration capacity. The results further showed that the diabetic sera profoundly abolished the chemotactic and migration behavior of EPCs as compared to parallel group (*p *= 0.0001), indicating that the diabetic sera presumably contained some factors which declined EPCs migration capacity in response to FGF stimulation (Figure 3C). 

*The functional uptake of Ac-LDL was abolished following diabetic exposure of hEPCs*

The analysis of immunofluorescence samples and flow cytometric assays revealed a decline in strength of Ac-LDL uptake after being-exposed with diabetic sera (Figure 4A-C). We precisely defined that the percentage of Dil-Ac-LDL positive cells decreased during 7-day incubation of hEPC with diabetic serum (*p* = 0.006). Based on these results, it was well-established that the EC progenitor′s potencies were significantly faded out to metabolize functional serum LDL mediated by Lipoprotein lipase.

*Total content of extracellular exosome content diminished by diabetic sera*

The quantitative assessment of released exosomes in terms of acetylcholine esterase activity showed an abrogated fusion of intracellular formed exosomes with cell membrane (Figure 5A). According to our results, the total content of released exosomes was significantly hindered as compared to normal sera-treated hEPCs over a course of seven days (*p* = 0.001). This means that cells exosome secretion capacity could be affected in diabetic condition. 

*The biogenesis of exosome was dominantly influenced in EPCs under diabetic condition*

We further investigated whether diabetic sera exposition of EPC could precisely impair the dynamic kinetics of the intracellular exosome, thereby three candidate genes, CD63, Alix, and Rab27a, involving in exosome trafficking were selected and monitored after seven days of incubation time. Quantitative real-time PCR analysis of cells in both groups, either in normal or diabetic conditions, showed a converged decrease in the expression of CD63, Alix, and Rab27a expression diabetic-exposed EPCs as compared to healthy non-diabetic sera (Figure 5B). Therefore, it was conclude that diabetic condition aberrantly diyregulated the expression of chief genes interfering in biogenesis, transfer, and release of exosome. As a result, we concluded that the exosome-mediated paracrine activity of EPCs could be hindered after being-exposed in diabetic condition. 

## Discussion

Uncontrolled alterations and impairment in the function of ECs and their progeny function have been previously documented due to the pathophysiology of type 2 diabetes mellitus (2). We here addressed the contributory effect of human diabetic sera on hEPCs pre-expanded over a course of seven days with focusing on paracrine properties mediated by dynamic kinetics of exosome. Three candidate important genes governing the exosome trafficking nominated as CD63-Alix-Rab27a axis were also monitored. 

Here, we showed that the diabetic sera induced the cytotoxic effect of hEPCs mainly via triggering the necrosis. It was already known that a great body of machinery underlying mechanisms participated in diabetic conditions of both type I or II that could induce adverse effects by triggering necrotic and apoptotic changes (20, 21). For example, Ardestani *et al,* defined an accelerated apoptosis via a newly signaling pathways modulated by mammalian sterile 20-like kinase-1 and converged up-relation of Bcl-2 in beta cells (22). In addition, raised levels of intracellular ionized calcium and advanced glycation end products (AGEs) coincided with sequential arrays of Caspase-3, iNOS over-activity and *etc*. which acted by NF-kB/IL-1β, IFN-γ/STAT-1 and MAPK signaling pathways in different typified cells (23). Additionally, by the elevation of blood viscosity during hyperglycemic conditions, it was acclaimed that worsened oxygen deprivation coupled with ATP deficiency in turn initiated coagulative necrosis of vascular niche and surrounding tissues (24). Chen *et al* acclaimed that 4-day incubation with high glucose condition enhanced EPC senescence via decreasing the amount of bioavailable nitric oxide, eNOS, FoxO1, and Akt phosphorylation (25). It worth noting that 7-day incubation of EPCs with type 2 diabetic condition showed a shift from apoptotic change to necrotic cytotoxic effect as seen in our current experiment. Noticeably, both time- and dose-dependent cytotoxic changes previously reported in hyperglycemic-primed EPCs (26). 

The migration capacity of diabetic sera-treated EPCs was also impaired in response to FGF stimulation. Many authorities outlined enormous underlying mechanisms to be related to unwillingness of EPCs toward different stimuli (27). Both type 1 and 2 conditions impaired the EPCs plasticity with cytoskeleton disarrangement in response to SDF-1α. Albumin bound AGE in combination with aberrant modulation of CXCR4/PI3K/Akt/eNOS signaling pathways could entirely lessen the EPCs mobility as well (28, 29). In addition, 48-h pre-incubation of EPCs with micro-vesicles originated from diabetic mice impaired their normal function (30). 

A reduction of approximately more than 50% in dynamics of circulating CD45^dim^CD34^+^KDR^+^ EPCs mobilization approved already in patients with pre-diabetic and diabetic status (9). Churdchomjan and co-workers found an inverse correlation with the number of circulating EPCs and elevation of glucose and HbA_1_c (26). In addition to a reduced pool of diabetic bone marrow hematopoietic and endothelial lineages, insufficient juxtacrine cell to cell interaction of EPCs with various cells, altered integrin profile and adhesion capacity to matrix molecules such as uncontrolled glycation of Arg-Gly-Asp (RGD) motif in fibronectin structure affected further extra- and intra-vasation properties (31, 32). Concurrent to taken *in-vitro* tubulogenesis results, numerous researches have been also confirmed a prohibitory effect of hyperglycemic environment on EPCs compartment in tube-like structures (33). One possible mechanistic inhibitory effect of diabetic-exposed EPCs is due to an over-activated Notch-1/Dll-4/Jag-1 signaling pathway (34)**. **Another striking concurrent adverse feature of diabetic condition on the Ac-LDL uptake of EPCs could be presumably related to a high level of lipids and inverse correlation of increased TNF-α factor, lessened statins level with simultaneous abrogated lipoprotein lipase activity (35). 

Our study also shed lights on paracrine secretion capacity of EPCs on healthy and diabetic subjects exerted by exosomes; a typified kind of micro-secretory vesicles. The amount of exosome released diminished into supernatant space by 7-day treatment with diabetic sera. Lower values in terms of exosome biogenesis, trafficking and secretion compared to normal conditions were also evident via manifestation of down-regulated pattern in set of CD63, Alix and Rab27a genes in response to diabetic-stimulated condition. Based on enormous different literature, CD63, Alix, and Rab27a genes actively participate in sequential steps of exosomes biogenesis, trafficking and abscission, respectively (13, 17). Of interest, a significant knowledge gap exists to decipher more comprehensive underlying mechanism in the different milieu. To our knowledge, although either beneficial or detrimental effects of diabetic conditions have been described on exosome secretion capacity for various cell types, but no informative study has been done so far on EPCs field (36). Collected data from different investigation must be however interpreted carefully. For example, some authorities acclaimed that some kind of cellular insults triggered an inducible release of exosome instead of constitutive form (37-39). In one study, the circulating levels of platelet- and EC-derived micro-vesicles were assayed through a type-1 and -2 diabetes mellitus disorders (40). For instance, Sabatier and co-workers reported a heightened level of micro-vesicles from two distinct cells type along with Annexin-V positive micro-vesicles in type 1 diabetes sera, but no momentous results observed in sera of second type of diabetes mellitus individuals (40). Chen *et al*. found a relevant increasing level of EPC micro-vesicles during 2 days priming with diabetic sera as compared normal sera exposed cells (30). 

One presumably potential reason for opposite results taken across current and different experiments might be due to incubation period and cell type and the varying concentrations of applied. We here strongly confirmed an adverse effect of diabetic on EPCs exosome secretion capacity through 7-day incubation. It was previously acclaimed that *in-vitro* 72-h incubation of beta cells with high glucose and fatty acid concentrations, mimicking chronic glucolipotoxic conditions, attributed to endoplasmic reticulum stress, reduction of cellular ATP, inositol 3 phosphate and cytoplasmic calcium content, which leaded to a decline in insulin granule docking to the plasma membrane (41). In addition, a deregulated manner of the IGF-1/Akt/mammalian target of rapamycin (mTOR) participating in exosome secretion could be happened during the onset of type 2 diabetes (42). Another possible mechanism in exosome biogenesis and dynamic secretion may be related to ceramide flow from endoplasmic reticulum to Golgi apparatus in diabetic glucolipotoxicity which is augmented by an unregulated status of sphingolipid biosynthesis, CERT-mediated ceramide transport, and decline in the phospho-Akt levels, and increased protein phosphorylation (43). It was previously well-established that maturation of reticulocyte into mature red blood cells decreases the potency of exosome secretion (44). It also could be hypothesized that an accelerated senesce of diabetic EPCs bona fide happened with a simultaneous decline in exosome secretion (45). In line with the down-regulated status of three genes, CD63, Alix and Rab27a, participating in exosome secretion, Olofsson *et al* revealed the small GTPase, Rab27a down-regulation and abrogation in releasable fusion of formed micro-vesicles to the plasma membrane (46). Additionally, we also notified that the biogenesis and MVB-related exosome formation and exosome trafficking concurrently suppressed in glucolipotoxicity-induced condition. 
